# Comparison of theoretical and experimental values for plant uptake of pesticide from soil

**DOI:** 10.1371/journal.pone.0172254

**Published:** 2017-02-17

**Authors:** Jeong-In Hwang, Sung-Eun Lee, Jang-Eok Kim

**Affiliations:** School of Applied Biosciences, Kyungpook National University, Daegu, Korea; MJP Rohilkhand University, INDIA

## Abstract

Pesticides that persist in soils may be taken up by the roots of plants. One way to assess plant uptake is to theoretically predict the extent of plant uptake using a mathematical model. In this study, a model was developed to predict plant uptake of pesticide residues in soils using various parameters, such as pesticide mobility within soil, plant transpiration stream, root–soil transfer rate, plant growth, and pesticide dissipation in either soils or plants. The accuracy of the model was evaluated by comparing the modeled concentrations with measured uptake concentrations of chlorpyrifos (CP) in lettuce, grown on treated soils with concentrations of approximately 10 and 20 mg kg^-1^ CP. Measured concentrations of CP in lettuce at 21, 30, and 40 d after planting were between the 5^th^ and 95^th^ percentiles of model variation. A high correlation coefficient of > 0.97 between modeled and measured concentrations was found. Coefficients of variation of mean factors to residual errors were between 25.3 and 48.2%. Overall, modeling results matched the experimental results well. Therefore, this plant uptake model could be used as an assessment tool to predict the extent of plant uptake of pesticide residues in soils.

## Introduction

Many studies have shown that theoretical prediction from mathematical models can assess the extent of plant uptake of hazardous substances, such as heavy metals and organic pollutants persistent in soil [1–6]. However, unlike heavy metals, it is difficult to develop a plant uptake model for organic chemicals, as their dissipation behaviors might be consistent or variable, depending on environmental conditions. Hence, plant uptake models tend to show low availability for organic contaminants. Nevertheless, regulatory authorities in several countries have utilized plant uptake models as a chemical exposure assessment tool, for examples, Soil Screening Levels (SSLs) in USA, Contaminated Land Exposure Assessment (CLEA) in UK, and CSOIL in Netherlands [7–9].

Plant uptake models for pesticides that persist in soils are based mainly on a bioconcentration factor (BCF) that indicates the ratio of pesticide concentrations in the plant and soil [10]. Shone and Wood [11] introduced the root concentration factor (RCF) to demonstrate the relationship between concentrations of pesticides in root and soil solutions. Thereafter, they defined the transpiration stream concentration factor (TSCF) that indicates the ratio of pesticides transferred from soil solution into the xylem sap of the plant [11,12]. Briggs *et al*. [13] found a high correlation between RCF or TSCF factors and octanol–water partition coefficients (*K*_ow_) of pesticides, and these correlations have been mainly considered in studies predicting the dynamic plant uptake of non- and weak-electrolytes as well as acids and bases [3,13].

TSCF has frequently been used in studies of the plant uptake model [14–16]. Charles [17] indicated that TSCF is an important concept for predicting the fraction of pesticides transferred from the roots to various compartments of the plant, such as stems, leaves, and fruits, by the transpiration stream. Moreover, RCF has been occasionally used to simulate the uptake and translocation of pesticides in fruit trees that have very thick fine roots [18]. Unusually, Felizeter *et al*. [19] used the RCF to model the uptake of acidic chemicals by lettuces, grown under hydroponic conditions.

When modeling the dynamic plant uptake of pesticides persistent in soil, it is necessary to consider adsorption and dissipation interactions between pesticide and soil [20]. These interactions are closely related to the mobility and persistency of pesticides in soil. Strong adsorption of pesticides on soil particles can result in lower soil mobility and reduced plant uptake, and more persistent pesticides may provide opportunities for more consistent uptake by plant roots. Our previous study showed that both the adsorption and dissipation interactions can be used as major parameters to model the residual magnitudes of pesticides in soils [20].

Dissipation of pesticides in plant is also a necessary consideration for modeling the fractions of reduction after uptake by the plant [6]. Dissipation routes of pesticides in plants may include removals by the plant metabolism and reversed translocation from roots to soil. In addition, the dilution of pesticide concentration during plant growth can contribute to dissipating pesticides in plants [21–24]. Juraske *et al*. [15] modeled the extent of plant uptake using a parameter that described the fraction of pesticide that is reflected back from roots to soil, opposite to the transpiration stream.

The organophosphorus insecticide chlorpyrifos (*O*,*O*-diethyl *O*-3,5,6-trichloro-2-pyridyl phosphorothioate, CP) has been widely used to control various pests occurred in agricultural industry [25]. Although half-lives of CP in soils are variable in the range of 1–100 d [26], the three chlorines in its structure can result in long-term persistence of CP in soils [20,27]. In addition, the low water solubility (1.4 mg L^-1^) and high log *K*_ow_ (4.7) values of CP may result in insignificant mobility in soil [28–30]. Therefore, it is likely that CP residue in arable soils is exposed to plant uptake, presenting a safety issue with final agricultural products.

This study aimed to develop a plant uptake model of CP from soil using parameters such as pesticide mobility in soil, plant transpiration stream, root–soil transfer rate, plant growth, and pesticide dissipation in either soils or plants. The accuracy of the developed model was statistically assessed by comparing the modeled estimates with measurements obtained from field experiments.

## Materials and methods

### Development of plant uptake model

#### Model approach

Pesticides in soils undergo either adsorption on soil particles and organic matters or dissipation by biotic and/or abiotic factors. Pesticide residues that do not undergo adsorption of dissipation in soil are present in soil solution, after which they are absorbed by plant roots with water uptake system. Hereafter, a fraction of absorbed pesticide is reflected back to the soil by the soil–root advection interaction and the resistance of waxy material (Casparian strip), covering the endodermis of the root [17]. Pesticide residues crossing the endodermis are transferred to aerial parts of the plant through xylem vessel. Downward translocation through the phloem of the plant cannot be included for non-ionic chemicals, such as pesticides, as there is no mechanism that they can retain them in phloem sap [1,31]. Finally, the uptake concentration of pesticides in plants decreases over time with the metabolism and growth of the plant. Taken together, the sequential steps for developing the dynamic plant uptake model in this study were: the dissipation and migration of pesticide in soil; pesticide residues in soil solution; root uptake of pesticide by the transpiration stream; and decrease in pesticide concentration in the plant.

#### Concentration in soil solution

Modeling of plant uptake of pesticide residues in soils was initiated at the point immediately after crops were planted. Based on our previous study [20], the concentration of pesticide in soil at time *t* (*C*_e_(*t*)) was calculated as the concentration in soil solution (Eq 1):

$${\text{C}}_{\text{e}}\left(t\right)=C_{0}\times {\left(1/2\right)}^{t/T}/K_{\text{d}}$$ (1)

where *C*_0_ is the initial pesticide concentration in soil (μg g^-1^), *T* is the half-life of pesticide in the soil (days), and *K*_d_ is the distribution coefficient of pesticide in soil and soil solution.

#### Pesticide concentration in the plant

The transpiration stream concentration factor (TSCF), which represents the ratio of pesticide concentrations in plant xylem sap and soil solution, is described by Charles [17] and Juraske *et al*. [15]. TSCF of non-ionic pesticide was estimated using eq 2:

$$\text{TSCF}=0.756\times \text{exp}\left[-{\left(\text{log}K_{\text{ow}}-2.50\right)}^{2}/2.58\right]$$ (2)

where *K*_ow_ is the octanol-water distribution coefficient of pesticide, which is obtained using the eq 3, described by Sabljić *et al*. [32]:

$$\text{log}K_{\text{oc}}=0.81\times \text{log}K_{\text{ow}}+0.1$$ (3)

where *K*_oc_ is the partition coefficient between organic carbon and water. TSCF corresponds to the fraction of pesticide absorbed into the plant with the transpiration stream [15]. However, to allow for the fraction reflected back by the endodermis in roots, transfer rate from soil solution to the plant (*k*_s-p_) was specified in eq 4:

$$k_{\text{s}-\text{p}}=Q_{\text{w}}\times \left(1-\text{TSCF}\right)$$ (4)

where *Q*_w_ is the plant transpiration stream (m^3^ day ^-1^) and the reflected fraction is expressed as ‘1 -TSCF’. A fraction of pesticide absorbed by the plant can be removed by plant. Removal rate of pesticide from the plant (*k*_rp_), based on first order kinetics, was described with eq 5:

$$k_{\text{rp}}=\text{ln}\left(2\right)/T_{\text{p}}$$ (5)

where *T*_p_ is the half-life of pesticide in plant. The time-dependent change in plant weight, representing pesticide concentration diluted by plant growth, (*M*_p_(*t*)) was calculated using eq 6 (*t* > 0):

$$\text{log}M_{\text{p}}\left(t\right)=I_{\text{g}}\times \text{exp}\left[k_{\text{g}}\times \text{log}t\right]$$ (6)

where *I*_g_ and *k*_g_ indicate the logarithmical plant weight at initial time 0, and a plant growth constant, respectively. The final plant uptake model developed in this study was specified by eq 7:

$$C_{\text{p}}\left(t\right)=\left\{C_{\text{e}}\left(t\right)\times k_{\text{s}-\text{p}}\times \left(1-\text{exp}\left[-k_{\text{rp}}\times t\right]\right)\right\}/\left(k_{\text{rp}}\times M_{\text{p}}\left(t\right)\right)$$ (7)

### Uptake experiments

#### Field trials

Three-weeks-old lettuce plug seedlings (Red Lollo Rosa cultivar) were purchased from a commercial plant nursery in Changnyeong, Korea. Uptake experiments were conducted in a greenhouse (2,092 m^2^ in area) on a lettuce farm in Waegwan (WG), Korea, between April 15 and May 25, 2014. Commercial CP (25% of wettable powder), diluted with 5 L of water was sprayed on one experimental plot (*n* = 3) of 100 × 500 cm using a shoulder-type compression sprayer, equipped with a 1-mm nozzle (KS-10-3, Kwang Sung Co., Daejeon, Korea); no additional pesticides were sprayed throughout the experimental period. Concentrations of CP were approximately 10 and 20 mg kg^-1^, corresponding to the low (LC) and high concentration (HC) treatments, respectively. Treated soils were homogenized to a depth of 10 cm and aged for 12 h before planting lettuce plug seedlings. The seedlings were planted at intervals of 10 cm. Water was supplied to the seedlings at a rate of 1.7 L/h for 20 min, every 5 d using an overhead sprinkler system. Conditions in the greenhouse were maintained at 23.1 ± 3.13°C with 60.3 ± 4.74% humidity. A control experimental plot treated with pesticide, but with no plants was prepared at the same time.

#### Preparation of plant and soil samples

Leaves and roots of lettuce plants (*n* = 20) were harvested from each experimental plot at 21, 30, and 40 d after the pesticide treatment. Roots were rinsed with running water to remove soil residues and lightly wiped with paper towel. Lettuce weights were measured at each sampling to obtain *M*_p_(*t*) values. Lettuce plants were divided into two compartments, roots and leaves that were individually homogenized using a grinder and stored in -20°C freezer (GC- 124HGFP, LG Electronics Inc., Seoul, Korea) prior to pesticide residue analysis.

Soil samples were collected from each experimental plot at 0 (12 h), 7, 14, 21, and 40 d after pesticide treatment, air-dried for 5 d, and passed through a 2-mm sieve. A portion of the samples was used to analyze of soil properties (S1 Table). Water content of soil samples was measured by comparing the change in weight from 24 h of oven-drying, and used to correct the residual concentration of CP in soil.

#### Pesticide residue analysis

CP residues from soil and lettuce samples, weighing 10 g each, were extracted with 80 mL of acetone. Soil samples were shaken at 200 rpm for 30 min using a shaking incubator (Vision Scientific Co., Ltd., Daejeon, Korea), and lettuce samples were homogenized at 12,000 rpm for 3 min using a grinder (AM-7, Nihonseiki Kaisha Ltd., Tokyo, Japan). Extracts of each sample were filtered through a Büchner funnel lined with filter paper (Whatman No. 2, Buckinghamshire, UK) and transferred into a separatory funnel containing 500 mL water, 50 mL of saturated sodium chloride solution, and 50 mL of methylene chloride. The funnel was shaken vigorously, and the organic solvent fraction was collected after dehydration with anhydrous sodium sulfate. The extraction step was repeated with the remaining residue in the funnel and a further 50 mL of methylene chloride. Organic solvent extracts were combined in one flask and concentrated using a rotary vacuum evaporator (Laborota 4000, Heidolph Instrument GmbH & Co., Schwabach, Germany) at 40°C. To purify, concentrates were re-dissolved with 10 mL of *n*-hexane and loaded into a glass column (16 mm i.d., 30 cm height), packed with 10 g of Florisil. Impurities in samples were removed with 60 mL *n*-hexane, and the fraction of pesticide was eluted with 60 mL of ethyl acetate/*n*-hexane (95/5, v/v). The final eluate was evaporated, dissolved with 2mL of acetone, and analyzed using a gas chromatography-mass spectrometer (GC-MS; Shimadzu GC 2010 equipped with a GC-MS QP-2010 Plus, Kyoto, Japan). All analysis of CP residues in soil and lettuce samples were based on a matrix matched calibration (MMC) method. S1 File describes the quality control procedure for the abovementioned analysis, MMC method, and analytical conditions of GC-MS.

The half-life (*T*) of CP in soil was calculated with the residual data, assuming the first order exponential dissipation (Eq 8):

$$T=\text{ln}\left(2\right)/k_{deg}$$ (8)

### Laboratory experiments

To obtain the *K*_d_ value, adsorption experiments of CP in soil followed procedures described previously [20]. Transpiration stream (*Q*_w_) of lettuce was measured using a potometer, which can record the volume of water uptake in plant. All methods for laboratory experiments are summarized in S1 File. The *K*_d_ and *Q*_w_ values obtained from these experiments were used as parameters in the model.

### Assessment of model accuracy

Accuracy of the plant uptake model was assessed by statistically comparing between modeled and measured concentrations. Based on the model assessment method by Juraske *et al*. [5], we evaluated the mean of the correlation coefficients (*R*^2^) and the error of residuals (ER), known as the standard deviation of the log of residuals between the modeled and measured values.

### Estimated CP concentration in leaves

The ratio of CP concentrations in leaves to those in the whole plant (*R*_L/W_) was calculated using data from the uptake experiments (Eq 9):

$$R_{\text{L}/\text{W}}=C_{\text{L}}/C_{\text{W}}$$ (9)

where *C*_L_ is CP concentration in leaves (mg kg^-1^), and *C*_W_ is CP concentration in the whole plant (mg kg^-1^). The *R*_L/W_ values were used to estimate the CP concentrations in lettuce leaves from the modeled results.

## Results and discussion

### Quality control

Total ion chromatogram and mass spectrum of CP identified by GC-MS are shown in S1 Fig. The mass spectrum of CP shared > 93% similarities with the mass spectral library data provided by the National Institute of Standards and Technology (NITS). The most intensive target ions (m/z 258 and 314) were used for the selected ion monitoring (SIM) analysis, which were free from fragment ions that appeared in blank soil and lettuce samples. Chromatograms for recovery tests are shown in S2 Fig. Peaks of CP in soil and lettuce that spiked at a concentration of 1.0 mg kg^-1^ showed clear shape and selectivity. In addition, there were no interfering substances in CP residue analysis. The linearity of calibration curves in the MMC method was acceptable, with correlation coefficients of >0.99. The minimum detectable amount (MDA) and limits of quantitation (LOQs) of CP were 0.1 ng and 0.02 mg kg^-1^, respectively. Recovery rates of CP that spiked at concentrations of 0.2 and 1.0 mg kg^-1^ in each sample were satisfactory at 88.1–93.5%, and relative standard deviations (RSD) were <7.2% (S2 Table). Therefore, this method of pesticide residue analysis was able to determine the CP residues from soil and lettuce samples.

### Adsorption and dissipation in soil

S3 Fig shows the adsorption behaviors of CP in WG soil. Adsorption of CP in the soil reached equilibrium after 24 h (S3a Fig), and the adsorption isotherm at that time was dependent on the treatment concentrations of CP (S3b Fig), showing a type C curve, as described by Giles *et al*. [33]. The *K*_d_ values of CP in the soil, calculated from the adsorption isotherm was 82.1 mL g^-1^ and ranged from 13.4 to 1862 mL g^-1^, as reported by Moore *et al*. [34].

Dissipation patterns of CP in soils collected from field experiments are illustrated in S4 Fig. Actual concentrations of CP in soils determined instrumentally at time 0 were 15.2 and 24.9 mg kg^-1^ in the LC and HC treatments, respectively, which were slightly higher than the nominal concentrations of 10 and 20 mg kg^-1^ that were expected when treating the pesticide. However, these actual concentrations were sufficient to determine the concentration dependency of pesticide uptake in lettuce. Concentrations of CP in soil at time 0 were used as an initial soil exposure concentration (*C*_0_) for plant uptake model. Soil half-lives (*T*) of CP in the LC and HC treatments were 17.2 and 7.9 d, respectively, and the dissipation rate was approximately twice as fast in the HC treatment as in the LC treatment. The faster dissipation of CP in the HC treatment implies less opportunity for root uptake of CP. Experimental values of adsorption (*K*_d_) and dissipation (*T*) were used as parameters in the plant uptake model.

### Uptake experiment

Measured uptake concentrations of CP in lettuce are shown in Table 1. In the LC treatment, 0.9 mg kg^-1^ of CP residue was absorbed from soil by lettuce after 21 d of growth, which decreased by 0.2 mg kg^-1^ (77.8%) after 40 d. Uptake concentration of CP in lettuce in the HC treatment was similar, at 0.8 mg kg^-1^ after 21 d of growth and declining to 0.1 mg kg^-1^ (87.5%) at the final sampling time. These results show that the extent of uptake of CP from soil by lettuce was not dependent on the concentration of pesticide in the soil.

**Table 1:** Uptake amount of chlorpyrifos (CP) from the contaminated soil by lettuce.

			Residual amount^a^ (mg/kg)		
			Compartment of lettuce		
Pesticide	Treated level (mg kg^-1^)	Time (day)	Leaf	Root	Whole
CP	10	21	0.5 ± 0.05	7.5 ± 0.66	0.9 ± 0.07
		30	0.4 ± 0.03	5.6 ± 0.38	0.6 ± 0.01
		40	0.1 ± 0.00	1.8 ± 0.12	0.2 ± 0.01
	20	21	0.8 ± 0.05	2.1 ± 0.14	0.8 ± 0.04
		30	0.3 ± 0.02	3.6 ± 0.05	0.5 ± 0.02
		40	0.1 ± 0.00	0.4 ± 0.00	0.1 ± 0.00

^a^ Mean of triplication ± SD

**Fig 1:**
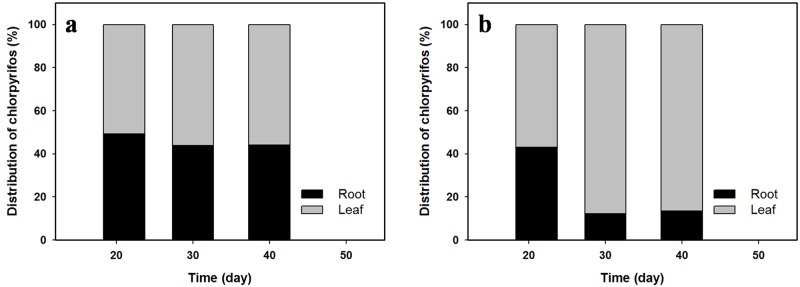
Time-dependent distributions of chlorpyrifos (CP) between roots and leaves of lettuce grown in soil contaminated with concentrations of (a) 10 and (b) 20 mg kg^-1^ CP.

Distribution patterns of CP in each compartment of lettuce differed between the LC and HC treatments. In the LC treatment, 50.9–56.1% of the uptake amount was constantly present in leaves throughout the experimental period (Fig 1a). However, the distribution rate in the HC treatment was 56.9% at 21 d of growth period and increased to 86.0% after 40 d of growth (Fig 1b). These results show that the significant amounts of CP can be transferred from roots to edible leaf parts, which may be due to the active growth of leaf parts during the experimental period (S5 Fig). Similar to our findings, Jeon *et al*. [35] reported the high concentrations (1.36–4.71 mg kg^-1^) of boscalid and chlorfenapyr from soil in Korean cabbage leaves. The *T*_p_ values in the LC and HC treatments were calculated at 8.7 and 6.3 d, respectively, and were used as model parameters to describe the removal rate of CP in lettuce.

### Comparison between modeled and measured data

Parameters of the plant uptake model are listed in Table 2. Most of the parameters were obtained from the laboratory experiments, except for half-life values of CP in soil and lettuce. Logarithmical correlation equations between the weight and growth time of lettuce were satisfactory, with correlation coefficients of >0.99, and the *I*_g_ and *K*_g_ constants in the equations were used as model parameters to calculate *M*_p_(*t*) values.

**Table 2:** Model parameters used for predicting root uptake of CP by lettuce.

		Value	
		Nominal treatment	
Input parameter (abbreviation)	Unit	10	20
Initial soil exposure concentration (*C*_0_)	mg kg^-1^	15.2	24.9
Half-life in soil (*T*)	day	17.2	7.9
Half-life in plant (*T*_p_)	day	8.7	8.0
Soil-water distribution coefficient (*K*_d_)	mL g^-1^	82.1	82.1
Organic carbon-water partition coefficient (*K*_oc_)	-	2218.9	2218.9
Octanol-water partition coefficient (*K*_ow_)	-	1.02×10^4^	1.02×10^4^
Transpiration stream (*Q*_w_)	mL day^-1^	46.8	46.8
Logarithmical initial plant weight (*I*_g_)	g	0.3062	0.3092
Plant growth constant (*K*_g_)	-	1.1020	1.2031

**Fig 2:**
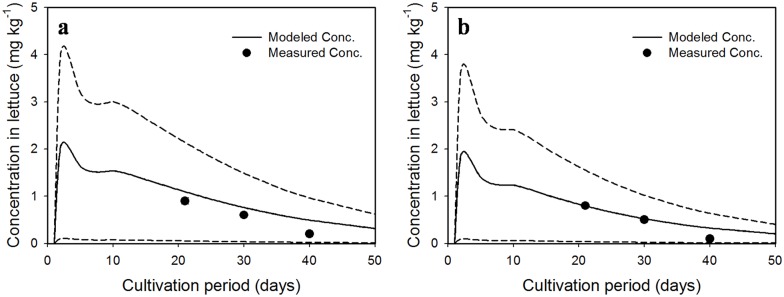
Uptake amounts of CP in lettuce grown in soils treated with concentrations of (a) 10 and (b) 20 mg kg^-1^ CP over the cultivation period. Means of measured concentrations (*n* = 3) are represented by *closed dots* (*error bars* denote standard deviations). *Solid lines* indicate modeled concentrations, while *dotted lines* display the 5^th^ and 95^th^ percentiles of model variation.

Uptake concentrations modeled using these parameters were compared with concentrations measured in the uptake experiments. As shown in Fig 2, the modeled concentration of CP in lettuce was the highest at 3 d of growth for both LC and HC treatments, due to the small weight of lettuce and high residual concentration of CP in soil. Thereafter, they decreased constantly with the increasing weight of lettuce and dissipation of CP in soil. Interestingly, all modeled concentrations in the HC treatment at 40 d were slightly lower than those modeled in the LC treatment, which may be attributed to the faster degradation rate of CP in HC-treated soils than in LC-treated soils. Similar to the modeled results, the measured uptake concentrations of CP during the uptake experiments were slightly lower in the HC treatment than in the LC treatment.

All measured concentrations were between the 5^th^ and 95^th^ percentiles of model variation. Although we found a large deviation (64%) between modeled and measured concentrations in both treatments at 40 d of growth, the mean value of deviations was acceptable at 27.9%, which is lower than the error value of 36% reported in another study [1]. Therefore, the modeled concentrations corresponded well to the measured concentrations.

### Model accuracy assessment

**Fig 3:**
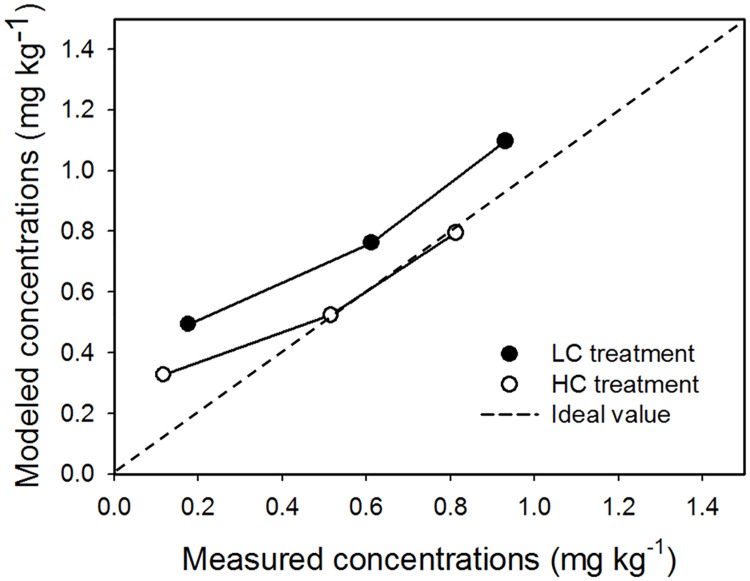
Comparison between modeled and measured uptake amounts of CP from soil by lettuce (*dotted line* is the ideal relation between both values).

The model accuracy assessment is presented in Fig 3. The correlation between modeled and measured concentrations was high, with *R*^2^ values of 0.97 to 0.98, and the ratio between both concentrations was close to an ideal value of 1. Mean ER value between modeled and measured concentrations was 0.18 in the LC treatment, corresponding to a mean factor of -0.70. In contrast, the mean value of ER in the HC treatment was 0.72, corresponding to a mean factor of -1.49. Herein, positive or negative factor values, which represent the mean of the log value of residuals between modeled and measured values, depend on whether the residual value is >1 [20]. Coefficients of variation (CV) of factors to ERs ranged from 25.3 to 58.2% and were higher than error values of 19–22.6% reported in other modeling studies [1,15,20].

Variations between modeled and measured concentrations may result from the absence of other influential parameters, such as the desorption and leaching of pesticide in soil, landscape of the field, environmental weather, and contact area between plant roots and soil. In other studies of plant uptake models for hazardous substances, researchers have tried to include such parameters in their models [36–39]. However, there is no complete plant uptake model that satisfies all parameters that are relevant to the plant–soil system.

### Estimated concentration in edible leaf parts

**Fig 4:**
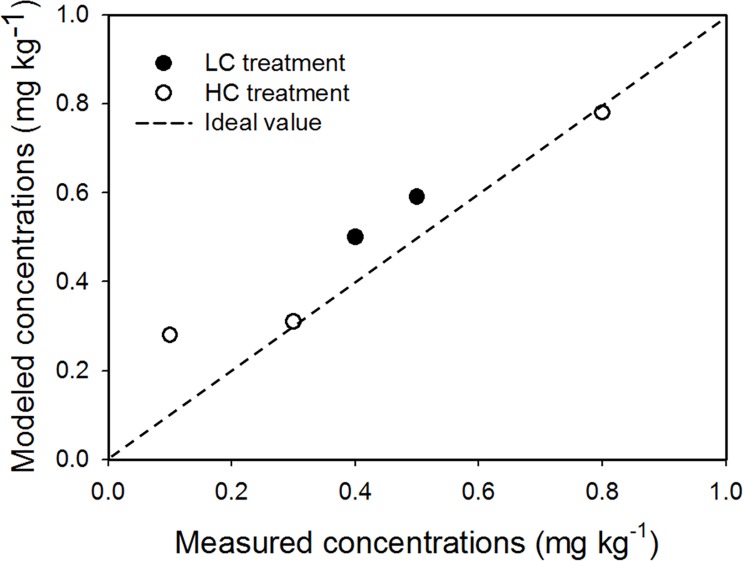
Correlation between modeled and measured concentrations of CP in leaves of lettuce (*dotted line* is the ideal relation between both values).

Based on the residual data analyzed from the uptake experiments (Table 1), the values of *R*_L/W_, which is the ratio of concentration in leaves and the whole plant, were calculated (S3 Table). Values of *R*_L/M_ were between 0.54 and 0.98, and were used to estimate the modeled concentrations of CP in leaves. Fig 4 show that the estimated concentrations of CP in lettuce leaves matched the experimentally-measured concentrations well, with a mean deviation of 27.9%, although the estimated CP concentrations at 40 d of growth deviated from those measured by approximately 64%.

Estimated CP concentrations in leaves were slightly higher in the HC treatment than in the LC treatment, similar to the uptake experiments. Estimated concentrations decreased slightly over time, with faster rates of decreasing CP in leaves estimated in the HC treatment. For both LC and HC treatments, the estimated concentrations ranged from 0.28 to 0.78 mg kg^-1^ during the sampling period of 21–40 d (S3 Table), similar to the concentrations measured in the uptake experiments (0.1–0.8 mg kg^-1^). All estimated and measured concentrations of CP in lettuce leaves exceeded the maximum residue limit (MRL) of 0.1 mg kg^-1^ [40]. Exceeding the MRL demonstrates that cultivating lettuce in soils contaminated with CP at concentrations of >2 mg kg^-1^ may lead to the production of unsafe lettuce at harvest.

In conclusion, our results suggest that the plant uptake model developed in this study could be used as a mathematical assessment tool to predict plant uptake of pesticides that persist in soils. In addition, further studies should be conducted to identify more effective parameters to improve the current low accuracy of plant uptake models.

## Supporting information

S1 Fig Total ion chromatogram (a) and mass spectrum (b) of CP analyzed using GC-MS. (TIF)

S2 Fig Chromatograms for recovery tests of CP in soil (a, b) and whole plant of lettuce (c, d), spiked at a concentration of 1.0 mg kg^-1^ (a, c—spiked samples; b, d—non-spiked samples). (TIF)

S3 Fig Adsorption kinetics (a) and isotherms (b) of CP on tested soils (*error bar* is the standard deviation of the triplicate measurement). (TIF)

S4 Fig Dissipation behavior of CP in the tested soil (*error bar* is the standard deviation of the triplicate measurement). (TIF)

S5 Fig Change in length (a) and weight (b) of lettuce parts during the field experiments. (TIF)

S1 File Laboratory experiment methods. (DOCX)

S1 Table Physicochemical properties of the soils used for adsorption tests. (DOCX)

S2 Table Recoveries of CP in soil and each compartment of lettuce. (DOCX)

S3 Table Estimated concentrations of CP in leaf parts, calculated using the *R*_L/W_ values. (DOCX)
