# Supplementary figures and images for: Comparison of theoretical and experimental values for plant uptake of pesticide from soil
Source: PLoS One. 2017 Feb 17;12(2):e0172254. doi: 10.1371/journal.pone.0172254 (PMC5315371; doi:10.1371/journal.pone.0172254)

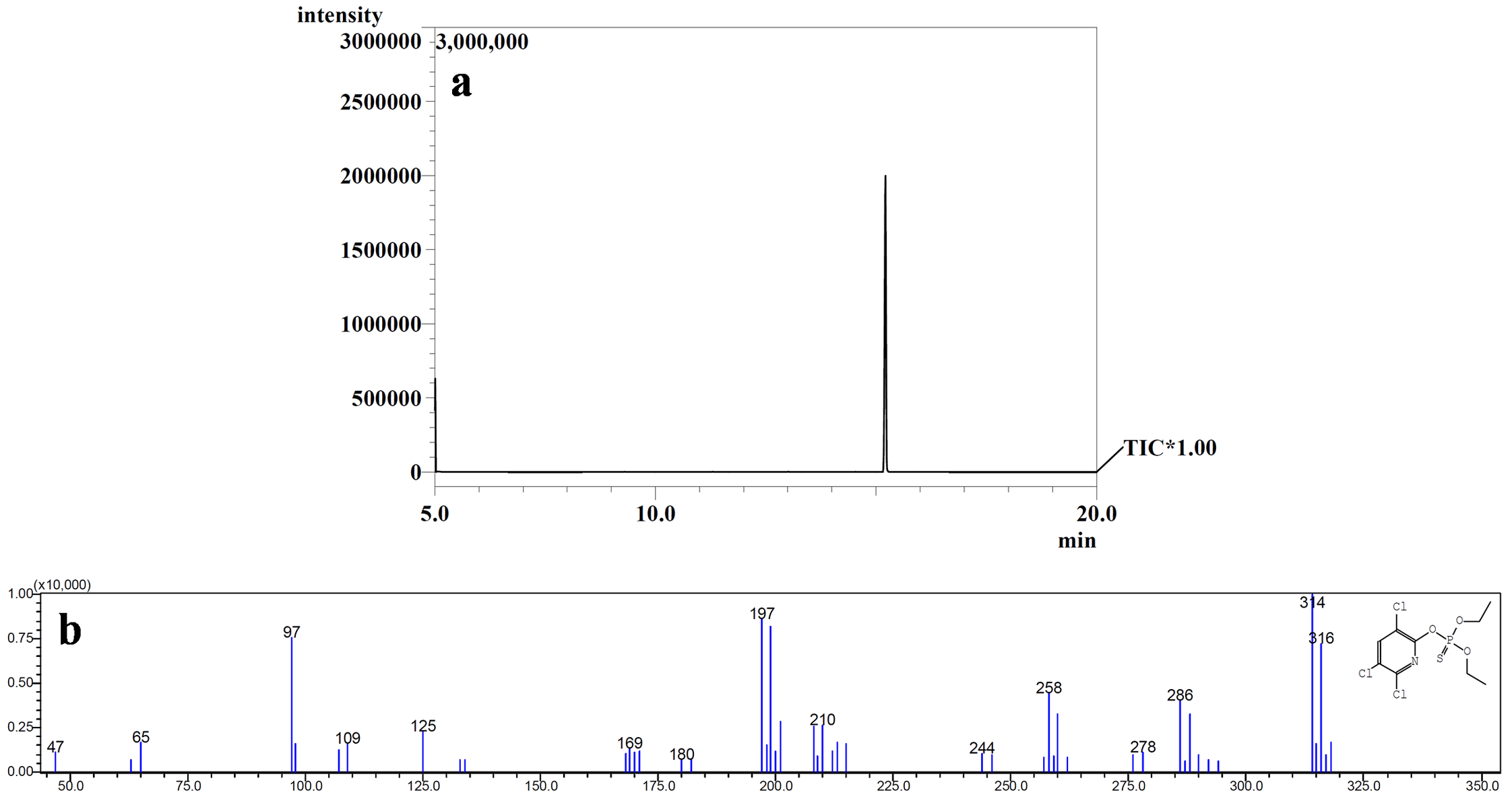

Supplement: S1 Fig — (TIF) [file pone.0172254.s001.tif]

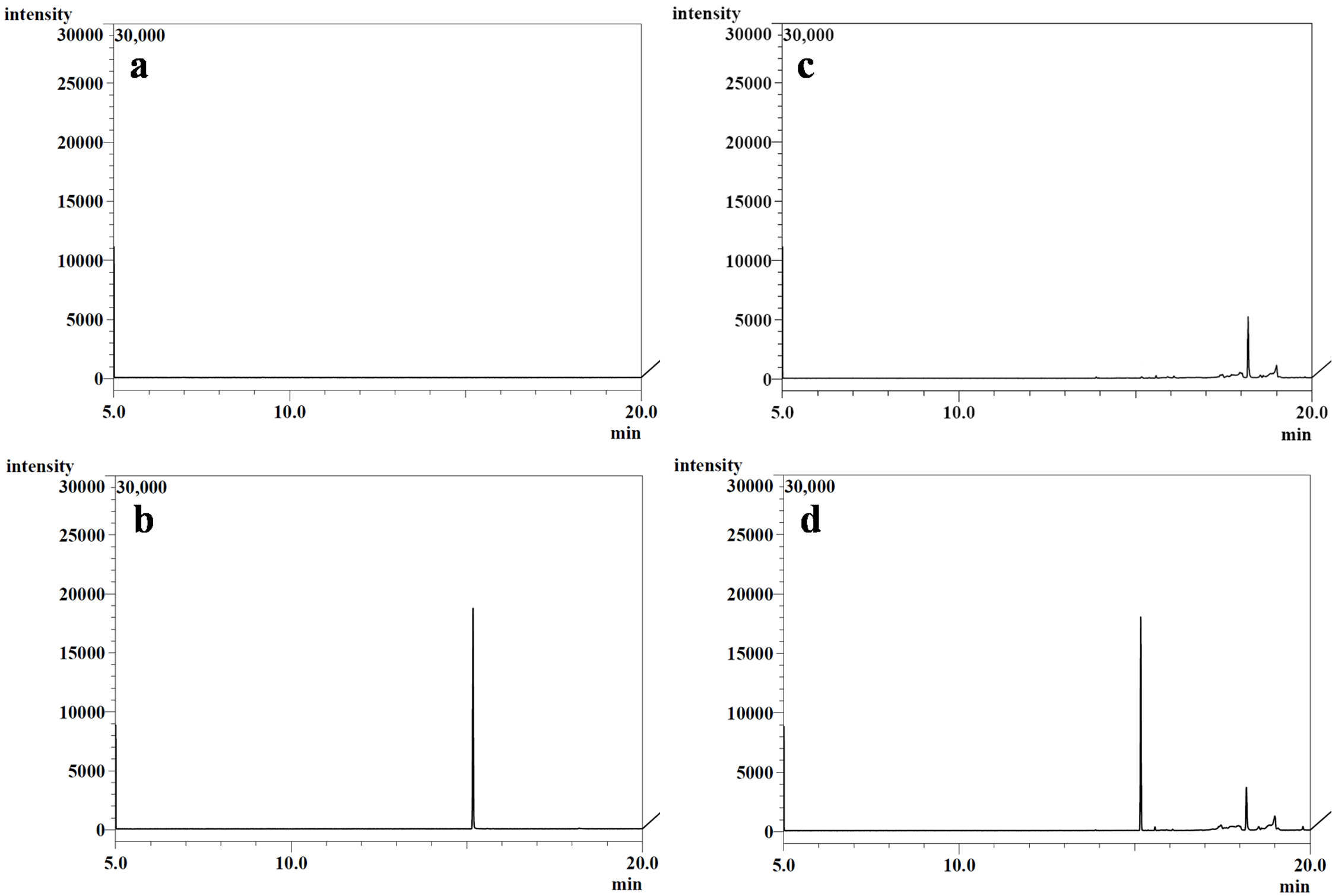

Supplement: S2 Fig — (TIF) [file pone.0172254.s002.tif]

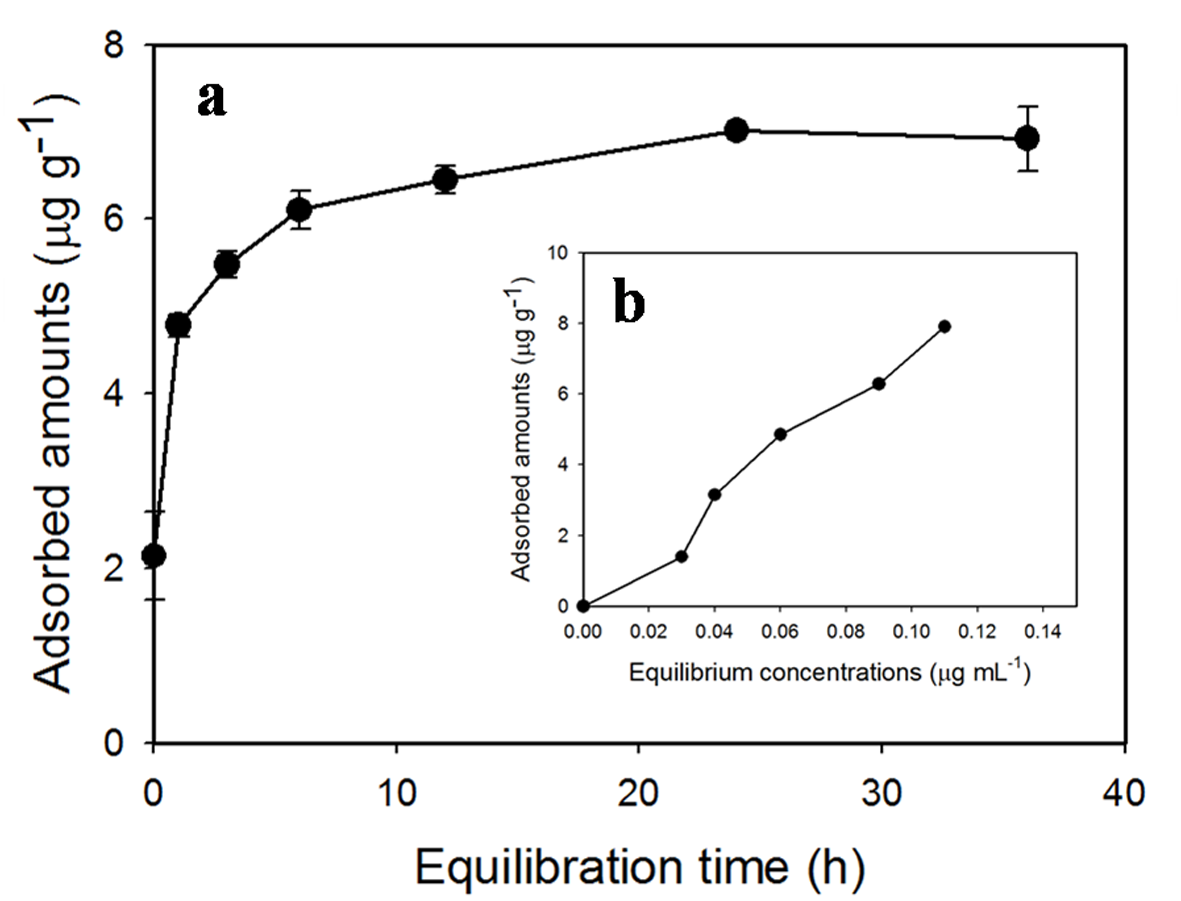

Supplement: S3 Fig — (TIF) [file pone.0172254.s003.tif]

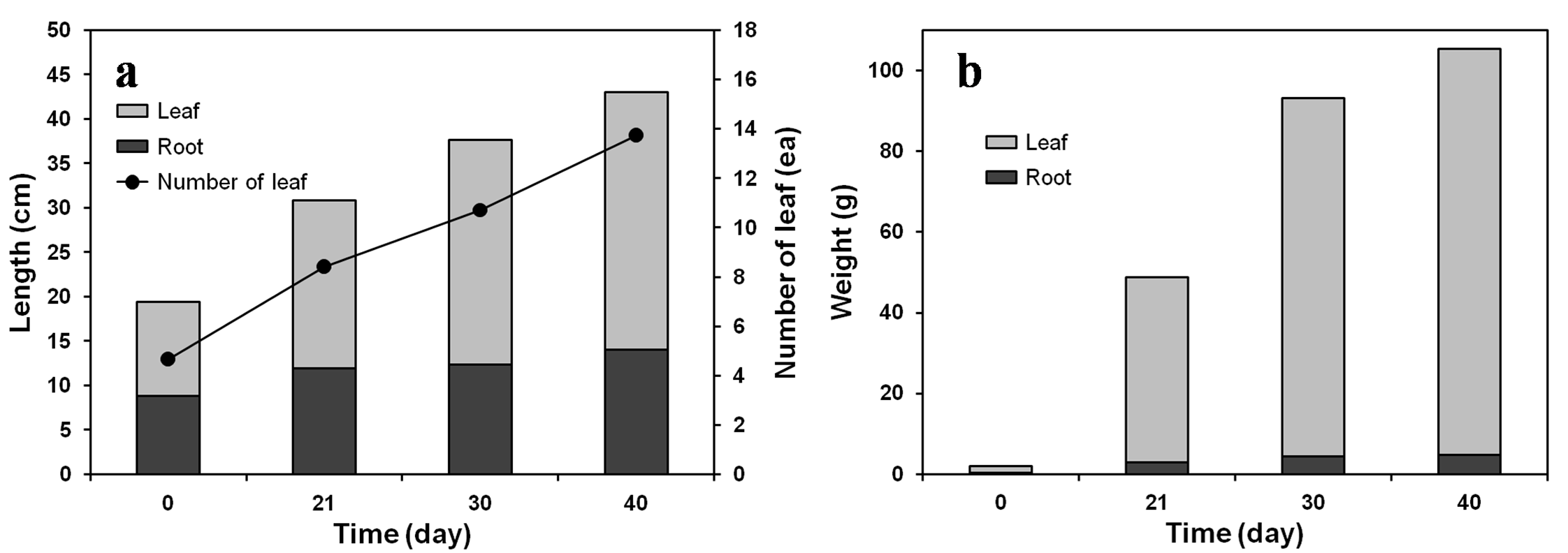

Supplement: S5 Fig — (TIF) [file pone.0172254.s005.tif]
