# Supplementary material for: Comparison of theoretical and experimental values for plant uptake of pesticide from soil
Source: PLoS One. 2017 Feb 17;12(2):e0172254. doi: 10.1371/journal.pone.0172254 (PMC5315371; doi:10.1371/journal.pone.0172254)
Supplement: S1 File — (DOCX) [file pone.0172254.s006.docx]

**Laboratory experiment**

**Adsorption test**

All adsorption experiments of CP in soil were conducted following a batch equilibrium method proposed by OECD (OECD, 2000). The CP-free soil collected from field trials were air-dried in the shade and sifted to a 2-mm size. The working solution of CP standard (99.7 % purity, Dow AgroSciences, Indianapolis, IN) was prepared at a concentration of 0.5 μg mL^-1^ in 0.01 M CaCl_2_ solution containing 1 % acetone and treated to 1 g of soil sample in a glass centrifuging tube at soil to solution ratio of 1:20 (w/v). To find the adsorption equilibrium time, the pesticide-treated tubes were shaken at 80 rpm in the shaking incubator for 0, 1, 3, 6, 12, 24, and 36 h at 25 ± 2°C in the dark. On the other hand, the CP adsorption in soil by a function of concentration was tested at the equilibrium time. The tested concentrations of CP in soil were 0.0, 0.1, 0.2, 0.3, 0.4, and 0.5 μg mL^-1^. All experiments were conducted with control (without soil) and blank (without pesticide) samples.

The samples collected from the shaking incubator were centrifuged at 3,000 rpm for 15 min. Fifteen milliliters of the supernatant was transferred into a separatory funnel and shaken vigorously with 15 mL of methylene chloride. The organic solvent layer was dehydrated through approximately 5.0 g of anhydrous sodium sulfate into an evaporating flask. The remaining part was extracted again with the same extraction step, and the extracts were combined. The combined extract was evaporated at 40°C using a rotary vacuum evaporator and re-dissolved with 2 mL acetone to analyze using the GC-MS.

All graphs of the adsorption isotherms were drawn using SigmaPlot 10.0 statistical software. The *K*_d_ values were calculated as the ratio between pesticide concentrations in soil and its solution based on a following equation.

*K*_d_ = *C*_s_ / *C*_e_

**Instrumental analysis conditions**

A GC-MS instrument was used to determine CP residues in the soil and lettuce samples. The injection port of instrument was maintained at 260°C, and 1 μL of each sample was injected into the port with no split mode. Temperatures of the interface and ion source box were set up to 300 and 200°C, respectively. The temperature of column oven equipped with a DB-5MS capillary column (30 m × 0.25 mm i.d. × 0.25 μm film thickness, J&W Scientific, Folsom, CA)was programmed as following conditions; initially held at 100°C for 2 min, raised by 10°C/min to 280°C, and then maintained for 6 min isothermally. The target ions of m/z 258 and 314 obtained from GC-MS mass spectra were used for the selective ion monitoring (SIM) analysis of CP in the samples.

**Quality control**

The quality control (QC) of the pesticide residue analysis method used in this study was tested in line with an assessing method proposed by US Environmental Protection Agency (U.S. EPA, 1994). The working standard solution of CP was prepared at known concentrations in acetone. To obtain the MMC solution, the working solution was added into each blank sample subjected to the pesticide residue analysis with no pesticide treatment using the previously described analytical method. The MMC solutions were prepared at the concentrations of 0.1, 0.2, 0.5, 1.0, 2.0, 5.0, and 10.0 μg mL^-1^. The limits of quantitation (LOQs) for each sample were calculated based on the minimum detectable amount (MDA) of CP in the GC-MS, and the linearity of MMC curves was checked on every sample analysis. To test the recoveries of CP in soil and plant sample, CP working solution was treated on each sample, containing no pesticide, at concentrations of 0.2 and 1.0 μg g^-1^, and they were analyzed, in triplicates, to determine the CP residues from them. At the same time, control samples prepared with no CP treatment were analyzed to identify CP from matrix. The method validation criteria for analysis of pesticide residues were followed to guidelines on good laboratory practice in pesticide residue analysis proposed by CODEX Alimentarius Committees (Codex Alimentarius Commission, 2010).

**Transpiration stream measurement of lettuce**

A part of lettuce samples collected from field trials was used to measure the transpiration stream (*Q*_w_). The root part of lettuce was cut right before measuring, and the cutting section of leaves was put at an end of potometer, equipped with a graduated pipette on the opposite end. The inside of potometer was fully filled with water, and the site connected with leaves of lettuce was sealed using a rubber cap. The water volume consumed by lettuce was recorded from the graduation of pipette every 30 seconds for 30 min. The mean value of measurements was used as the *Q*_w_ parameter value.

**References**

1. Codex Alimentarius Commission. Guidelines on good laboratory practice in pesticide residue analysis (CAC/GL 40-1993). 2010. pp. 1-36.
2. Organization for Economic Cooperation and Development (OECD). Test No. 106: Adsorption-desorption using a batch equilibrium method. In: OECD guidelines for the testing of chemicals. Paris France: OECD; 2000.
3. United States Environmental Protection Agency (U.S. EPA). Methods for assessing the toxicity of sediment-associated contaminants with estuarine and marine amphipods (EPA 600/R-94/025). 1994. pp. 1-140.
