# Supplementary material for: Comparison of theoretical and experimental values for plant uptake of pesticide from soil
Source: PLoS One. 2017 Feb 17;12(2):e0172254. doi: 10.1371/journal.pone.0172254 (PMC5315371; doi:10.1371/journal.pone.0172254)
Supplement: S1 Table — (DOCX) [file pone.0172254.s007.docx]

S1 Table. Physicochemical properties of the soils used for adsorption tests

| Sampling  site | Soil  texture | pH  (1:5) | Exchangeable cations  (cmol_c_ kg^-1^) | | | OM^a)^  (g kg^-1^) | EC^b)^  (dS m^-1^) | Particle  distribution (%) | | |
| --- | --- | --- | --- | --- | --- | --- | --- | --- | --- | --- |
|  |  |  | K | Ca | Mg |  |  | Sand | Silt | Clay |
| WG^c)^ | Sandy  loam | 6.3  ± 0.1 | 1.4  ± 0.6 | 16.4  ± 0.2 | 4.3  ± 0.4 | 37.0  ± 1.3 | 9.2  ± 0.4 | 55.0  ± 2.4 | 40.3  ± 0.8 | 4.7  ± 0.4 |

^a)^ OM, organic matter; ^b)^ EC, electrical conductivity; ^c)^ WG, soil sampled in Waegwan region
