# Supplementary material for: Comparison of theoretical and experimental values for plant uptake of pesticide from soil
Source: PLoS One. 2017 Feb 17;12(2):e0172254. doi: 10.1371/journal.pone.0172254 (PMC5315371; doi:10.1371/journal.pone.0172254)
Supplement: S2 Table — (DOCX) [file pone.0172254.s008.docx]

S2 Table. Recoveries of CP in soil and each compartment of lettuce

| Pesticide | Treated level  (mg kg^-1^) | Recovery rate^a)^ (%) ± SD | | |  |
| --- | --- | --- | --- | --- | --- |
|  |  | Leaf | Root | Soil | |
| CP | 0.2 | 91.2 ± 4.2 | 90.2 ± 3.2 | 91.6 ± 6.6 | |
|  | 1.0 | 88.1 ± 6.1 | 93.5 ± 4.4 | 89.3 ± 6.3 | |

^a)^ Mean of triplication ± SD
