# Supplementary material for: Comparison of theoretical and experimental values for plant uptake of pesticide from soil
Source: PLoS One. 2017 Feb 17;12(2):e0172254. doi: 10.1371/journal.pone.0172254 (PMC5315371; doi:10.1371/journal.pone.0172254)
Supplement: S3 Table — (DOCX) [file pone.0172254.s009.docx]

Table S3. Estimated concentrations of CP in leaf parts, calculated using the *R*_L/W_ values

| Days after transplanting (d) | *R*_L/W_^a)^ | | Estimated concentration  in leaf part (mg kg^-1^) | |
| --- | --- | --- | --- | --- |
|  | LC^b)^ | HC^c)^ | LC^b)^ | HC^c)^ |
| 21 | 0.54 | 0.98 | 0.59 | 0.78 |
| 30 | 0.65 | 0.58 | 0.50 | 0.31 |
| 40 | 0.57 | 0.86 | 0.28 | 0.28 |

^a)^ Ratio of concentrations between leaf part of lettuce and its whole plant

^b)^ LC, low concentration treatment

^c)^ HC, high concentration treatment
